# The Influence of the COVID-19 Pandemic on the Imports and Exports in China, Japan, and South Korea

**DOI:** 10.3389/fpubh.2021.682693

**Published:** 2021-07-12

**Authors:** Panpan Wei, Cheng Jin, Chen Xu

**Affiliations:** ^1^Department of Asian Languages, Zhejiang Yuexiu University, Shaoxing, China; ^2^Department of Economics, Shaoxing University, Shaoxing, China

**Keywords:** COVID-19, import and export trade, China, Japan and South Korea, government intervention

## Abstract

In this paper, time-series and cross-country data spanning from January 2020 to December 2020 are adopted to empirically investigate the impact of the COVID-19 pandemic on exports and imports in China, Japan, and South Korea. In the models, industrial production, trade openness, government response (including monetary and fiscal intervention), and the pandemic impact of major trade partners are controlled. In addition, the three countries, China, Japan, and South Korea, are also estimated separately in consideration of the cross-country disparity. The results show that domestic epidemics in China, Japan, and South Korea have a non-significant (statistically significant) effect on imports, but are negatively correlated with exports in Japan; epidemics in major trading partners are negatively correlated with imports in Japan and positively correlated with exports in China and South Korea; and government intervention is positively correlated with imports in China and positively correlated with exports in China, Japan, and South Korea.

## Introduction

The global economy has suffered a severe shock since the outbreak of the COVID-19 pandemic. In April 2020, the World Trade Organization (WTO) predicted that under an optimistic scenario, global trade would shrink by 12.9% in 2020, while a pessimistic scenario predicted 32%. In October 2020, the WTO issued a newly revised forecast in light of the changing situation, projecting that global merchandise trade would fall by 9.2%. This epidemic has a long incubation period, a high transmission rate, and strict control over the gathering and movement of people. Therefore, under the new situation, it is expected that the impact of the epidemic on industry will be greater than that of SARS in 2003, and the impact on the restructuring and layout of domestic economy in China, Japan, and South Korea will be more profound. From the production point of view, the impact of the epidemic on labor-intensive industries such as textiles, garments, construction materials, toys, leather, and furniture manufacturing is greater. These industries are generally characterized by high labor and capital costs. In terms of exports, the epidemic had a greater impact on outward-oriented industries. Yiwen and Xiaomei (1) conducted a quantitative study based on historical data and found that the SARS epidemic significantly inhibited the exports of Chinese enterprises and the impact of the epidemic on exports would not recover in the short term, and there was also a certain negative impact in the long term. According to Zhen and Liwei (2), the current epidemic will have a serious impact on the Chinese manufacturing industry as a whole. China's printing industry, furniture and other manufacturing industries, textile and leather manufacturing industries, and automobile manufacturing industries have greater trade dependence and are therefore more vulnerable to the global chain of industries brought about by the epidemic. From the consumption point of view, epidemic prevention and control drives the pharmaceutical industry and non-contact industry market surge. Demand for medical protective gear, medical equipment, proprietary Chinese medicines, and anti-viral drugs appeared to increase (3). By April 2020, the epidemic was under control in China, therefore its imports and exports volume had been positive since June, growing 1.9% from a year earlier, which not only made China the only major economy in the world to achieve positive growth in merchandise trade but also pushed its share of global exports, climbing to a record high of 14.2%. As the world's third largest economy, although its import and export trade fell compared to the same period last year, Japan's exports to China grew significantly, increasing by 2.7% for the year. The proportion of Japan's exports to China rose to 22% of its total exports, making China the largest export destination. South Korea's share of total global trade was 3%, reaching an all-time high in 2011, and ranked ninth, the same as the previous year. With the epidemic under better control, China, Japan, and South Korea have become the “growth engines” of the Asian economy and even the world, therefore the value of studying their trades has increased. This research focuses on the following two main areas.

The first is the study of the impact of the COVID-19 pandemic on the trade and economy among China, Japan, and South Korea. Chinese scholars believe that the epidemic has brought new opportunities and challenges for China-Japan-ROK economic and trade cooperation, and many of them are optimistic about the future prospects of China-Japan-ROK cooperation. In an article written by Yongsheng (4), it is argued that as long as the global pandemic is controlled extremely effectively, China-Japan-ROK FTA negotiations are expected to succeed in the near future and the future prospects will be very broad. Yueju (5) not only mentions that the epidemic brought a considerable impact on the economic growth rate and economic and trade relations among China, Japan, and South Korea in the first quarter but also points out that the experience of cooperation against the epidemic, the willingness to recover the economy for China, Japan, and South Korea, and the rapid recovery of the Chinese economy will create more new opportunities for the recovery and development of economic and trade relations among the three countries and their FTA negotiations. Xiao and Yingda (6) summarize post epidemic China-Japan-ROK opportunities as: opportunities for cooperation against the epidemic, the willingness of the three countries to cooperate in economic and trade, the demand for development in high-tech fields, as well as the stability of the situation in northeast Asia. They summarize the challenges affecting the cooperation among the three countries as: racist rhetoric, anti-globalization thinking, unstable bilateral relations, industrial homogenization competition, deteriorating Sino-US relations, and the North/South Korean nuclear issue (6).

Unlike China, scholars in Japan and South Korea are less likely to study the impact of the epidemic on China, Japan, and South Korea as a whole. They focus on the impact of the epidemic on their own countries, on a particular country, or on the world economy as a whole. South Korean domestic scholars are more concerned about the impact of the epidemic on global value chains and their own economies. Dongchul (7), Sungok (8), and Yangmi (9), among others, express concern about damage to global value chains. Hyuntai and Dosook (10) focus their attention on China to propose three aspects of improving Sino-Korean economic relations, including postponing economic restructuring, developing a non-contact economy, and strengthening Sino-Korean economic cooperation, based on the analysis of the impact of the epidemic on the Chinese economy. Economic Laboratory (11) suggests that “reverse globalization and big government” is a global trend after the epidemic which will be a major challenge for the South Korean economy. The trend of de-Sinicization is expected to accelerate after the epidemic. Relative studies by Japanese scholars are few but those that do focus on China, the United States, Russia, North Korea, South Korea, and India. Kazuhiro (12) points out that “the South Korean economy, which has a high degree of external dependence, has suffered a huge blow due to the expansion of the worldwide epidemic infection. In particular, the high dependence on China of the South Korean economy has exposed its vulnerability. The South Korean government has tried to strengthen its economic ties with Association of Southeast Asian Nations (ASEAN) countries and India, but this alone is not enough to mitigate the risk of losing shares in the Chinese market. It is also in the national interest for South Korea to work to improve relations with Japan and to win over the Japanese market.” Eri and Kenji (13) argue that the epidemic has impacted the reconstruction of the Global Supply Chain (GSC) and it is important to take appropriate measures to manage global supply chain risk.

The second is specific research on the impact of the COVID-19 pandemic on import and export trade. Domestic Chinese scholars have focused on the impact of the epidemic on domestic import and export trade, and most of the relevant studies have been conducted on data from the early stage of the epidemic. By compiling and analyzing the import and export trade data between China and 249 countries or regions, Xiuyu (14) finds that from January to April 2020 the import and export trade between China and the ASEAN showed an upward trend compared to the same period last year. Some scholars also predict the impact of the epidemic on China's import and export trade based on the impact of major epidemic outbreaks on imports and exports in history. Based on the six public health emergencies of international concern (PHEIC) that WHO has declared since 2005, Qian and Yu (15) predicted that the country's exports would be about 15–20% lower in the first quarter; and about 15% lower in the second quarter. From a full-year perspective, along with the turnaround of the epidemic, exports will rapidly resume growth after the second quarter, and will be about 3–8% lower throughout the year than in 2019. Combining the data related to import and export from January to February after the epidemic, Jingan and Hailong (16), pointed out that despite the global character of the epidemic, it would not have a global impact on China's international economic influence, and the negative impact on China's import and export trade would only be a temporary phase ripple. According to Wonseok (17), the share of exports from Hubei in South Korea's imports from China is negligible (1.0% in 2018), and the impact on South Korean exports is expected to be small. However, compared to the SARS period (2003), South Korea's value chain to China has further deepened, and the negative impact on the South Korean economy will increase if the blunting of China's exports and consumption is prolonged. Fernando and Ana Maria (18) found that the impact of international trade of essential goods during the epidemic depends crucially on the countries' trade imbalances in essential goods. For example, net importers of these goods are relatively worse off during a pandemic than net exporters. The welfare losses of net importers are lower in a world with high trade barriers, while the reverse is the case for net exporters. Yet, once a pandemic arrives, net exporters of essential goods benefit from an increase in trade barriers, while net importers benefit from a decrease in them.

From the above studies, it can be seen that, compared with Chinese scholars, scholars from Japan and South Korea seldom study China, Japan, and South Korea as a whole, and there are even some de-Sinicization propositions and views in the Japanese and South Korean studies. In terms of research content, there are not many studies involving the impact of import and export trade, and most of them are about the impact on China's import and export trade, with no studies involving China, Japan, and South Korea yet. In terms of research time, most of the relevant studies appear in the time of the epidemic. In terms of research methodology, most of the studies on the impact of the epidemic are based on the historical situation of the epidemic or the data of the early stage of the epidemic, while impact factors outside the epidemic are not taken into consideration, which makes it difficult to accurately grasp the real situation of the impact of the epidemic. In order to overcome the shortcomings mentioned above, this thesis investigates the impact of the COVID-19 pandemic on China-Japan-South Korea economies and trade through the empirical analysis method based on the monthly changes of the pandemic in 2020, aiming to reveal the relationship between the COVID-19 pandemic and China-Japan-South Korea economies and trade. Then, this thesis outlines the change of import and export trade under the influence of the epidemic, and explores the regional economic and trade cooperation under the continuous downturn of the external market.

## Data and Methodology

This paper intends to study the impact of COVID-19 on China, Japan, and South Korea's international trade since its outbreak, covering the period from January 2020 to December 2020, largely dictated by the availability of the data on time span. Each country's import (henceforth IMPT) and export (henceforth EXPT) are measured by the growth rates compared to the same months of the previous year, which are drawn from the website of related government departments (detailed sources are shown in Table A1 in Appendix). Import and export are separately investigated to account for the differences in the impacts of COVID-19. The original data used to calculate the COVID-19 outbreak degree of the three countries (henceforth NIF), which is new infections per 10,000 people, sourced from WHO.

$$\begin{array}{l} NIF=Newinfectionspermonth\;\times 10,000\;/Totalpopulation \end{array}$$

The higher the NIF value is, the more serious the epidemic is in the corresponding country. In the estimation, to consider the possible impact of the country's main trade partners' epidemic outbreak, a variable of the epidemic outbreak degree of main trade partners (henceforth PNIF) are introduced with the same calculation method of NIF, the data of which are also sourced from WHO. Specifically, China, Japan, and South Korea's top 20 trade partners consist of their most international trade, with 64.8, 82.4, and 81% in import, and 66.7, 76.7, and 78.9% in export, respectively. Hence we mainly consider the epidemic situation of their top 20 trade partners in measuring PNIF, while these three country's main trade partners are also highly overlapped (countries are shown in Table A2 in Appendix).

Moreover, a country's government power, or government intervention (henceforth GovI) is measured by the ratio of government expenditure to the nation's economic size, following Nurudeen and Usman (19), as

$$\begin{array}{l} GovI_{it}=\frac{\overset{t}{\underset{j=1}{\sum }}GEX_{ij}}{GDP_{i}} \end{array}$$

Here, *GEX*_*ij*_ represents the government expenditure coping with the impact of the epidemic in country *i* and month *j*. The special government bond and special public expenditure confronting the epidemic are used to approximately measure government intervention (GEX). These data are drawn from the Ministry of Finance (China, Japan) and the Ministry of Economy & Finance (South Korea). As the effect of government fiscal expenditure is consistent and accumulative, we use the cumulant from month 1 to t to measure government intervention in month t. *GDP*_*i*_ is the whole year's (2020) gross domestic product in country I, used as a proxy of a country's size of economy.

In addition, as GDP at the month level lack data in these three countries and approximately 70% of their international trade in goods are industry products, therefore, we use the SP to represent the value of industrial production, and then the growth rate of industrial production (hereafter InP) is used as a proxy for economic output growth. Also, using

$$\begin{array}{l} TRO_{it}=\frac{IMPT_{it}\text{+}EXPT_{it}}{SP_{it}} \end{array}$$

to measure trade openness.

The data are obtained from China's National Bureau of Statistics and Organization for Economic Co-operation and Development (OECD) data. In the estimation, we consider four samples, which are China, Japan, South Korea, and these three northeast countries as a whole. Table 1 contains statistics summarized from the above major variables.

**Table 1:** Summary statistics of selected variables from January 2020 to December 2020.

	**Mean**	**Median**	**Maximum**	**Minimum**	**Std. dev**.	**Observations**
IMPT	−6.55	−5.5	12.70	−26.1	9.13	48
EXPT	−3.83	−3.00	20.60	−40.6	13.17	48
InP	−2.12	−0.54	7.30	−32.73	8.83	48
NIF	0.57	0.14	3.67	0.00	0.91	48
GovI	0.03	0.02	0.07	0.00	0.02	48
TRO	36.92	30.91	65.35	24.12	13.94	48
PNIF	11.38	8.35	40.17	0.00	10.79	48

*IMPT, EXPT, INP, NIF, TRO, PNIF, and GovI denote import, export, industry product, new infections, trade openness, main trade partners' new infections, and government intervention, respectively*.

### Correlation Analysis

Table 2 provides the bivariate association between variables.

**Table 2:** Correlation analysis.

	**IMPT**	**EXPT**	**InP**	**INF**	**GovI**	**TRO**	**PNI**
IMPT	1.00						
EXPT	0.49	1.00					
InP	0.36	0.86	1.00				
NIF	−0.18	−0.01	−0.16	1.00			
GovI	0.56	0.71	0.57	−0.12	1.00		
TRO	0.12	0.06	0.29	0.05	0.04	1.00	
PNI	0.34	0.67	0.40	0.29	0.81	−0.13	1.00

*IMPT, EXPT, INP, NIF, TRO, PNIF, and GovI denote import, export, industry product, new infections, trade openness, main trade partners' new infections, and government intervention, respectively*.

Based on the above theoretical background and data, the first time-series empirical regression takes the following linear form:

$$\begin{array}{l} \ln\;{\left(X\right)}_{i}=c+\alpha_{1}\ln\;{\left(InP\right)}_{i}+\alpha_{2}\ln\;{\left(InNIF\right)}_{i}+\overset{4}{\underset{j=3}{\sum }}\partial_{j}\ln\;{\left(InZ\right)}_{i}+\varepsilon_{i} \end{array}$$

Here, the subscripts i denotes countries; c is constant; α_j_ (j = 1,.,4) are the estimated coefficients of corresponding independent variables; X is import (IMPT) or export (EXPT); Z is also a set of explanatory variables including NIF and TRO; and ε is the error term. In the estimation, we add 45 to each number of IMPT & EXPT and add 35 to each number of Inp before the logarithm of them.

Then we consider the possible impact of the visible hand—government intervention (GovI), the empirical form is as follows:

$$\begin{array}{l} \ln{\left(X\right)}_{{}_{i}}=c+\alpha_{1}\ln{\left(InP\right)}_{{}_{i}}+\alpha_{2}\ln{\left(InNIF\right)}_{{}_{i}}+\alpha_{3}\ln{\left(InGovI\right)}_{{}_{i}}\\ \;+\sum_{j=4}^{5}\partial_{j}\ln{\left(InZ\right)}_{{}_{i}}+\varepsilon_{i} \end{array}$$

We also use panel data for empirical estimation as an overall analysis both in import (IMPT) and export (EXPT), compared to the previous time series estimation. Two typical models are used in the estimation, the fixed effect (FE) model and the random effect (RE) model, which use a fixed average value, an error term of time-series, and cross-section characteristics, respectively. The estimating equation is as follows:

$$\begin{array}{l} \ln{\left(X\right)}_{{}_{it}}=c+\alpha_{1}\ln{\left(InP\right)}_{{}_{it}}+\alpha_{2}\ln{\left(InNIF\right)}_{{}_{it}}+\alpha_{3}\ln{\left(InGovI\right)}_{{}_{it}}\\ \;+\sum_{j=4}^{5}\partial_{j}\ln{\left(InZ\right)}_{{}_{it}}+\varepsilon_{it}+\mu_{it} \end{array}$$

Here, the subscripts i and t also represent countries and months; except for the error term of ε, μ denotes the random unobservable country effects. The selection of an FE or RE model is determined by the Hausman test.

Hausman test.

H0: ε_*I*_ is not correlated with LnP, LnNIF, LnGovI, and InZ.
H1: ε_*I*_ is correlated with LnP, LnNIF, LnGovI, and InZ.

## Empirical Results

### Import

The estimated results of the import based on the time-series data are summarized in Table 3. Except for the sample of China, Japan, and South Korea, “The three” takes these three northeast countries as a whole. The difference between models (A) and (B), models (C) and (D), models (E) and (F), and models (G) and (H) is the inclusion of GovI as an explanatory variable.

**Table 3:** Estimated results on import.

	**China**		**Japan**		**South Korea**		**The Three**	
	**Model (A)**	**Model (B)**	**Model (C)**	**Model (D)**	**Model (E)**	**Model (F)**	**Model (G)**	**Model (H)**
C	0.72 (6.75)	3.80 (5.96)	12.56^*^ (5.52)	12.15^**^ (5.04)	−3.96 (4.78)	2.10 (5.41)	6.02 (7.62)	7.74 (7.91)
InP	−0.07 (0.12)	−0.09 (0.11)	0.48^*^ (0.23)	0.59^**^ (0.23)	1.49^**^ (0.57)	1.51^**^ (0.51)	−0.01 (0.23)	−0.05 (0.23)
NIF	0.08 (0.09)	0.07 (0.07)	−0.03 (0.10)	−0.02 (0.09)	0.02 (0.04)	0.04 (0.03)	0.11 (0.06)	0.07 (0.07)
TRO	1.04^*^ (1.94)	0.48^*^ (1.68)	−3.10 (1.55)	−3.47 (1.42)	0.55 (1.40)	−0.40 (1.39)	−0.61 (2.24)	−0.88 (2.28)
PNIF	0.05 (0.03)	−0.02 (0.04)	−0.00^*^ (0.08)	0.06 (0.08)	0.01 (0.04)	−0.20 (0.13)	0.01 (0.02)	−0.02 (0.04)
GovI		0.32^*^ (0.17)		−0.26 (0.16)		0.50 (0.31)		0.19 (0.20)
R^2^	0.44	0.65	0.67	0.76	0.83	0.88		0.45
Observations	12	12	12	12	12	12	12	12

*IMPT, EXPT, InP, NIF, TRO, PNIF, and GovI denote import, export, industry product, new infections, trade openness, main trade partners' new infections, and government intervention, respectively*.

Standard errors are in parentheses.

(**) *, and*

(*) *: significant at 5, and 10% level, respectively. C, constant term; R^2^, reliability squaring*.

The estimated coefficient of InP in models (A), (B), (C), and (D) are positive and statistically significant at a 5–10% significance level. It implies that a higher level of industry output growth tends to increase the growth rate of import in Japan and South Korea. This is probably due to the fact that an expansion in production leads to an increase in consumption capacity of goods abroad, as well as demand of the intermediate or raw materials for further production. However, the NIF coefficients and PNIF coefficients [except model (C)] are all statistically insignificant at the conventional significance level without model specifications. A plausible explanatory is that the time span covers just 12 months, limited by the fact that the outbreak of the epidemic began around January 2020. The negative impact of PNIF shown in model (C) is significant at the 10% significance level, albeit with a very tiny coefficient (0.00 after rounding). It means that the seriousness of the epidemic situation may slightly decrease Japan's import. As Japan is a highly industrialized open economy occupying the high end of the international value chain, the deterioration of its main partners' epidemic situation may affect its import through two ways: One is that it may directly decrease these countries' intermediate products' productivity which is needed for further production. The other can be that it decreases these countries' demand of Japan's goods which are produced from raw materials or intermediate products imported abroad.

Particularly, the GovI coefficient in model (B) is positively associated with IMPT and statistically significant at the conventional significance level, whereas they are statistically insignificant in model (D) and (H). It suggests that China's government intervention against the impact of COVID-19 has a positive effect on its import. A plausible expansion is that it stimulates China's investment and consumption which demand raw and processed materials and goods from abroad. The impact of TRO is positive in the sample of China (models A and B) and statistically significant, which is consistent with the existing literature as (20).

### Export

The estimated results of the export are summarized in Table 4, following the same approach as the previous results in import. It is found that a negative association exists between NIF and EXPT with the coefficients statistically significant in models (C) and (D), but they are statistically insignificant in the remaining models. It suggests that the control of the epidemic contributed to Japan's export. On the other hand, the coefficients of PNIF are positive and statistically significant in models (A) and (F), implying that China and South Korea's export growth rate is not only related to their own epidemic situation, but also their main trade partners'. That is, the more serious their partners' epidemic situation is, the higher export growth rate they have.

**Table 4:** Estimated results on export.

	**China**		**Japan**		**South Korea**		**The Three**	
	**Model (A)**	**Model (B)**	**Model (C)**	**Model (D)**	**Model (E)**	**Model (F)**	**Model (G)**	**Model (H)**
C	3.10 (2.85)	4.87^**^ (1.76)	−0.89 (3.94)	−0.51 (3.13)	−14.6 (8.21)	2.55 (6.69)	−1.09 (2.02)	−0.03 (1.27)
InP	0.90^***^ (0.05)	0.90^***^ (0.03)	1.08^***^ (0.17)	0.98^***^ (0.14)	1.08 (1.07)	1.13 (0.63)	0.96^***^ (0.06)	0.94^***^ (0.04)
NIF	0.10 (0.04)	0.09 (0.02)	−0.02^*^ (0.07)	−0.03^*^ (0.06)	0.00 (0.06)	0.05 (0.04)	0.11 (0.02)	0.08 (0.01)
TRO	−0.61 (0.82)	−0.93 (0.50)	0.25^*^ (1.10)	0.58^*^ (0.89)	3.50^*^ (2.62)	0.78 (1.71)	0.49 (0.59)	0.33 (0.36)
PNIF	0.04^***^ (0.01)	0.00 (0.01)	0.02 (0.05)	0.03 (0.05)	0.09 (0.07)	0.50^**^ (0.16)	0.01 (0.01)	0.01^*^ (0.01)
GovI		0.19^***^ (0.05)		0.23^*^ (0.10)		1.42^***^ (0.38)		0.12^**^ (0.03)
R^2^	0.67	0.99	0.92	0.96	0.73	0.92	0.98	0.99
Observations	12	12	12	12	12	12	12	12

*IMPT, EXPT, InP, NIF, TRO, PNIF, and GovI denote import, export, industry product, new infections, trade openness, main trade partners' new infections, and government intervention, respectively*.

*Standard errors are in parentheses*.

(***) *,*

(**) *, and*

(*) *: significant at 1, 5, and 10% level, respectively*.

*C, constant term; R^2^, reliability squaring*.

The coefficients of government intervention (GovI) on export growth rate (IMPT) are positive and statistically significant in all models. The results show that China, Japan, and South Korea governments' intervention against the epidemic contribute significantly to the recovery and re-booming of their export. InP and TRO show similar correlations with previous estimations in import.

In addition to the time-series estimation on the impact in each country, panel data consisting of these three countries are simultaneously analyzed to investigate the overall impact on the northeast countries as a whole, which to a certain extent can be used as a robust check. The estimation results are demonstrated in Table 5. It shows that the epidemic outbreak had a negative impact on the export of these northeast countries, whereas the estimated coefficients are statistically insignificant on the import. The results in model (A) and model (D) also show that the deterioration of the epidemic in their main trade partner has a negative association with the northeast countries' import and a positive association with their export, both of the coefficients are statistically significant at the conventional significance level. Moreover, in models (B) and (C), it also suggests that the impact of government intervention (GovI) is positive both on import and export. The very similar results from the panel data estimation reconfirm the previous investigation in Tables 3, 4.

**Table 5:** Estimated results of the north-east countries.

	**Import**		**Export**	
	**Model (A)**	**Model (B)**	**Model (C)**	**Model (D)**
C	2.84^***^ (0.32)	4.45^***^ (0.79)	1.29^***^ (0.26)	2.00^***^ (0.69)
InP	−0.12 (0.09)	−0.13 (0.08)	0.87^***^ (0.07)	0.86^***^ (0.07)
NIF	−0.08 (0.02)	−0.03 (0.03)	−0.01^*^ (0.01)	−0.01^*^ (0.03)
TRO	0.29^***^ (0.09)	0.08 (0.13)	−0.18 (0.07)	−0.28 (0.11)
PNIF	−0.04^*^ (0.05)	0.10 (0.08)	0.01 (0.04)	0.07^*^ (0.07)
GovI		0.21^**^ (0.10)		0.09^*^ (0.08)
X2 [*p*-value]	15.93 [0.00]	5 [0.01]	5 [0.03]	28.35 [0.00]
Estimation method	Fixed effect	Fixed effect	Fixed effect	Fixed effect
R^2^	76	0.81	0.95	0.96
Observations	36	36	36	36

*IMPT, EXPT, InP, NIF, TRO, PNIF, and GovI denote import, export, industry product, new infections, trade openness, main trade partners' new infections, and government intervention, respectively*.

*Standard errors are in parentheses*.

(***) *,*

(**) *, and*

(*) *: significant at 1, 5, and 10% level, respectively*.

*C, constant term; R^2^, reliability squaring*.

## Discussion

The domestic epidemic had no significant (statistically) impact on the imports of the three countries. In terms of exports, it is negatively correlated with Japan, for instance. The better the epidemic is controlled in Japan, the more favorable it is for exports. Although the epidemic in China, Japan, and South Korea was generally controllable in 2020, the epidemic in Japan was significantly higher than that in China (number of infected people: 95701, infection rate: 0.68 per 10,000) and South Korea (number of infected people: 48331, infection rate: 9.3 per 10,000) in terms of both the number of infected people (193071) and the infection rate (15.3 per 10,000).

For a long time, manufacturing and international trade have been the main drivers of economic growth in China, Japan, and South Korea. However, compared with goods trade, the three countries have maintained a long-term deficit in service trade and a small share of foreign trade, especially China, whose service trade accounts for only 14.6% of foreign trade. In addition, considering the differences of influencing elements between goods trade and service trade, the study of this thesis is currently only concerned with goods trade. Only a small portion of the foreign trade in goods of the three countries is made up of primary products, with most of them industrial ones. Therefore, our study can generally reflect the situation of international trade in manufacturing industries in China, Japan, and South Korea under the impact of the epidemic. This thesis does not address the extent of the impact of the epidemic on industries such as the service sector. Xian and Zifei (21) mention that “the biggest impact of the epidemic on the U.S. was mainly in the tertiary sector, especially the restaurant, airline, retail, and hotel industries were severely damaged, while the biggest impact on Chinese industries was not the tertiary sector as perceived, but on industry, which was down 9.6% in the first quarter of 2020, far outpacing the tertiary sector's 5.2%.”

Asia was the only region in the world to maintain positive growth (0.3%) in the volume of goods trade exports in 2020, and the role the three countries played as leading growth in Asian economies was an important content to examine in this thesis. Our related research will not stop there. In fact, we also prefer to study the problem from the perspective of the manufacturing industry. As time accumulates, certain available data spans will accumulate to meet our conditions for conducting regressions, and we will examine the impact of the epidemic on the manufacturing industry based on this thesis. For example, quarterly reports are available in the accounting statements of listed companies, and when a certain time span is accumulated, we intend to study the impact of the epidemic on the manufacturing industry from the aspect of manufacturing companies.

The epidemic in major trading partners is negatively correlated with Japan's imports, but the coefficient is small, implying that the impact of trading partners affects Japan's imports, but the effect is minor. On the export side, the severer the epidemic in Japan's major trading partners, the better China and South Korea's exports are. This is because their producing capacity declines due to the epidemic, with orders shifted to the two other countries, or the demand for the two countries' exports rises significantly. The epidemic kept recurring in many countries, some of the countries' factories were forced to stop production, the industry chain was broken, and some foreign trade orders from India, Bangladesh, Vietnam, and other countries were transferred to China. In addition, the demand for epidemic prevention materials such as masks, protective clothing, epidemic prevention drugs, and “home economy” products such as computers and household appliances from major trading partners also boosted the exports of both countries.

Government intervention is positively related to China's imports and also positively related to China, Japan, and South Korea's exports. It shows that government financial intervention is beneficial to the recovery of the country's international trade, especially in terms of exports. The government intervention in China has been particularly successful and has had a positive effect on imports and exports.

## Limitations and Prospects

Due to the relatively short period of time (1 year) since the onset of the epidemic, the time span of the relevant data can only be accurate to the monthly level, so there are only 12 observations in the time dimension of this study, resulting in a small number of study samples. And we have selected and illustrated only the results with a high degree of creditability in the description; this also limits the use of our estimation methods. With time ongoing, more observations can be obtained, allowing us to further process these data. For example, to analyze the cointegration of the time series data, and if the cointegration is inconsistent, we will try to use the ARDL or nonlinear-ARDL approach to analyze the endogeneity of the panel data. If there is endogeneity, we will try to analyze it by the GMM method. In addition, there are many other factors affecting the import and export trade, and this study only selected a few main variables during the epidemic period, i.e., the epidemic, trade openness, and government intervention. Considering the impact of the epidemic on import and export trade and commodity structure among China, Japan, and South Korea, we can increase the sample size and analyze other influencing factors in future studies.

## Conclusion

Industrial structure in China, Japan, and South Korea are highly similar, but there is heterogeneity in the impact of the epidemic on imports and exports in China, Japan, and South Korea. The epidemic stimulated the demand for medical supplies and boosted export growth, and government intervention had a positive impact on imports and exports.

## Data Availability Statement

The raw data supporting the conclusions of this article will be made available by the authors, without undue reservation.

## Author Contributions

Material preparation and data collection and analysis were performed by PW and CX. The first draft of the manuscript was written by PW and all authors commented on previous versions of the manuscript. All authors contributed to the study conception and design. All authors read and approved the final manuscript.

## Conflict of Interest

The authors declare that the research was conducted in the absence of any commercial or financial relationships that could be construed as a potential conflict of interest.
