# Supplementary material for: The Influence of the COVID-19 Pandemic on the Imports and Exports in China, Japan, and South Korea
Source: Front Public Health. 2021 Jul 12;9:682693. doi: 10.3389/fpubh.2021.682693 (PMC8336864; doi:10.3389/fpubh.2021.682693)
Supplement: Supplementary file 1 [file Data_Sheet_1.docx]

Appendix

Table A1. Data sources of the main variables

| Variables | China | Japan | South Korea |
| --- | --- | --- | --- |
| NIF,PNI | World Health Organization(WHO)  https://covid19.who.int/ | | |
| IMPT, EXPT | General Administration of Customs of China  http://www.customs.gov.cn/ | Ministry of Finance of Japan[www.mof.go.jp/](http://www.baidu.com/link?url=TqhOBJ7VMSX0PBlNsRRVVmLYT3TGD1C71rbLvTtg9vS) | Korea Customs Service  www.customs.go.kr/ |
| InP | National Bureau of Statistics of China  http://www.stats.gov.cn/ | Organization for Economic Co-operation and Development(OECD)  http://www.oecd.org/index.htm | |
| GEX | Ministry of Finance of China  http://gks.mof.gov.cn/ztztz/guozaiguanli/ | Ministry of Finance，Japan  https://www.mof.go.jp/ | [Ministry of Economy and Finance](https://english.moef.go.kr/) of Korea  www.moef.go.kr |

Table A2. Countries of top 20 trade partners in the sample

|  | China | Japan | South Korea | The three countries as a whole |
| --- | --- | --- | --- | --- |
| Main Trade partners |  | China | |  |
|  | Japan |  | Japan |  |
|  | South Korea, UK, Brazil | |  | UK, Brazil |
|  |  | Italy | |  |
|  |  | | Saudi Arabia, UAE | |
|  | Saudi Arabia |  |  |  |
|  |  | China | |  |
|  | USA, Canada, Germany, Netherlands, France, Russia, Australia, Mexico, Vietnam, Malaysia, Thailand, Philippines，Singapore, India，and Indonesia | | | |
